# Grem1 accelerates nucleus pulposus cell apoptosis and intervertebral disc degeneration by inhibiting TGF-β-mediated Smad2/3 phosphorylation

**DOI:** 10.1038/s12276-022-00753-9

**Published:** 2022-04-19

**Authors:** Shunlun Chen, Linchuan Lei, Zemin Li, Fan Chen, Yuming Huang, Guowei Jiang, Xingyu Guo, Zhuoyang Zhao, Hui Liu, Hua Wang, Caijun Liu, Zhaomin Zheng, Jianru Wang

**Affiliations:** 1 Department of Spine Surgery, The First Affiliated Hospital of Sun Yat-sen University, 510080 Guangzhou, China; 2 Department of Spine Surgery, The Third Affiliated Hospital of Guangzhou University of Traditional Chinese Medicine, 510378 Guangzhou, China; 3 Pain Research Center, Sun Yat-Sen University, 510080 Guangzhou, China

**Keywords:** Experimental models of disease, Biological therapy

## Abstract

Intervertebral disc degeneration (IVDD) is a main cause of low back pain, and inflammatory factors play key roles in its pathogenesis. Gremlin-1 (Grem1) was reported to induce an inflammatory response in other fields. This study aimed to investigate the mechanisms of Grem1 in the degenerative process of intervertebral discs. Dysregulated genes were determined by analyzing microarray profiles. The expression of Grem1 in 17 human disc samples (male:female = 9:8) and rat models (*n* = 5 each group) was measured by western blotting (WB), real-time quantitative PCR (RT-qPCR), and immunohistochemistry (IHC). The regulatory effects of Grem1 on apoptosis were examined using siRNAs, flow cytometry, immunofluorescence (IF), and WB. The therapeutic effect was evaluated by locally injecting specific Grem1 siRNA into IVDD rats. The expression of Grem1 was significantly increased in human degenerative intervertebral discs; furthermore, the expression of Grem1 positively correlated with the level of intervertebral disc degeneration. Grem1 was significantly overexpressed in tumor necrosis factor (TNF)-α-induced degenerative NP cells. Apoptosis in degenerative NP cells transfected with siRNA targeting Grem1 was significantly lower than that in the control group. Specific Grem1 siRNA markedly repressed the development of IVDD in surgery-induced IVDD rats. These results indicated that the expression of Grem1 was positively correlated with the severity of intervertebral disc degeneration, and Grem1 siRNA could inhibit Grem1-induced apoptosis and extracellular matrix alterations by mediating the TGF-β/Smad signaling pathway. This study may provide a therapeutic strategy for alleviating inflammation-induced apoptosis associated with intervertebral disc degeneration.

## Disc generation: Protein identified as potential therapeutic target

Gene expression profiling reveals an important factor underlying degeneration of the discs that connect and cushion individual vertebrae, a primary cause of lower back pain. This degeneration generally originates in the central portion of the disc known as the nucleus pulposus (NP). Researchers led by Jianru Wang and Zhaomin Zheng of the First Affiliated Hospital of Sun Yat-Sen University, Guangzhou, compared NP cells from healthy individuals with those from patients with disc degeneration. They observed strongly increased expression of a protein called Gremlin-1, which is critical to development and also associated with osteoarthritis. Gremlin-1 expression correlated strongly with degeneration and death in cultured NP cells, and the researchers identified key signaling pathways affected by this protein. Targeted inhibition of Gremlin-1 prevented tissue damage in a rat model of disc degeneration, highlighting a potential therapeutic opportunity.

## Introduction

Low back pain (LBP) is experienced by 11–84% of individuals worldwide during their lifetime^1,2^. Estimates suggest that LBP is the most common cause of disability and has heavy global economic and health burdens in low-income and middle-income countries^3,4^. Although the causes of LBP are complicated and multifactorial, intervertebral disc degeneration (IVDD) is the primary cause of LBP, and nucleus pulposus (NP) dysfunction is considered to be the initiating factor in IVDD^5,6^. An intervertebral disc (IVD) is composed of an inner gel-like NP and an outer fibrocartilaginous annulus fibrosus (AF), borders the upper and lower endplates and connects adjacent vertebral bodies^6^. An imbalance in anabolic and catabolic processes initiates a degenerative cascade in the disc, and the characteristic pathological changes include increased proinflammatory cytokines, loss of extracellular matrix (ECM), decreased numbers of nucleus pulposus cells (NPCs), the transition of cell phenotype, cell senescence and cell death^6–8^. Studies have shown that the inflammatory response in disc cells is a critical event during IVDD progression^7,8^. Our previous studies indicated that inflammation could change the microenvironment of NPCs, induce apoptosis or pyroptosis and ultimately lead to IVDD^9–11^.

Grem1, which encodes Gremlin-1, is an antagonist of bone morphogenic proteins (BMPs) and plays a critical role in embryogenesis, organ development, tissue differentiation, and bone formation^12,13^. Grem1 has also been reported to be involved in cancer, organ fibrosis and inflammation^14,15^. Studies have shown that Grem1 is significantly upregulated in human osteoarthritis and animal models of osteoarthritis^16–20^. Few studies have shown the expression of Grem1 in IVD cells, and the most abundant expression occurs in NPCs^21,22^. One study showed that Grem1 was downregulated in NPCs treated with extracellular vesicles^23^. However, there has been no study about the correlation of Grem1 expression with IVDD. Transforming growth factor-β (TGF-β) is a multifunctional cytokine and a crucial mediator that regulates cell differentiation, apoptosis, tissue fibrosis and migration, and TGF-β/Smad signaling is a canonical pathway for cell processes^24,25^. Studies have demonstrated that the TGF-β/Smad signaling pathway is involved in articular cartilage with osteoarthritis^26–28^. More importantly, studies have indicated that TGF-β signaling may play an important regulatory role in the process of IVD degeneration^29–31^. We hypothesized that Grem1 plays an important role in the progression of IVDD and that the TGF-β/Smad signaling pathway participates in the progression.

It remains unclear whether TGF-β1/Smad signaling is involved in the maintenance or destruction of IVDD. In this study, we identified Grem1 as one of the most significantly upregulated genes by comparing differentially expressed mRNAs in NP tissues from IVDD patients and normal controls via a systematic analysis of public microarray datasets of IVDD obtained from the Gene Expression Omnibus. Our hypothesis was confirmed by in vitro and in vivo experiments. Hence, further upstream and downstream pathways were examined, and the TGF-β/Smad signaling pathway was found to be significantly downregulated in Grem1-mediated IVDD.

Therefore, the objective of this study was to investigate the molecular mechanisms of Grem1 during the degenerative progression of IVD and its relationship with LBP.

## Materials and methods

### Patient samples

A total of 17 NP samples were collected from 17 patients (male:female = 9:8) with disc disease who underwent operations at the First Affiliated Hospital of Sun Yat-sen University (Guangzhou, China). Detailed information on the participants is listed in Table 1. Grade I and II discs were collected from fresh traumatic disc fracture or deformity cases, and Grade III, IV, and V discs were collected from degenerative disc disease cases. Routine T2-weighted magnetic resonance imaging scans of the lumbar spine and image analysis were performed before surgery. The degree of disc degeneration was analyzed by three independent observers according to the Pfirrmann classification. This study protocol was approved by the Medical Ethics Committee of The First Affiliated Hospital of Sun Yat-sen University, and written informed consent was obtained from each patient.

**Table 1:** Information on human disc samples from 17 patients.

Human disc samples	Sex	Age	level	Grade
1	M	15 y	T9/10	I
2	F	14 y	T8/9	I
3	M	17 y	T8/9	I
4	F	25 y	L3/4	II
5	M	31 y	L4/5	II
6	M	24 y	L2/3	II
7	F	45 y	L3/4	III
8	F	50 y	L3/4	III
9	F	37 y	L2/3	III
10	M	44 y	L4/5	III
11	M	67 y	L4/5	IV
12	F	59 y	L5/S1	IV
13	M	55 y	L3/4	IV
14	M	71 y	L3/4	IV
15	F	85 y	L4/5	V
16	F	81 y	L2/3	V
17	M	69 y	L3/4	V

*F* female, *M* male, *y* year.

### Isolation and three-dimensional culture of NP cells

Human NP cells were isolated by the methods described in our previous study^10^. Matrigel (BD Biosciences, San Jose, CA) was thawed at 4 °C overnight, and then Costar Transwell inserts (Corning, NY) were coated with 60 μL and incubated at 37 °C for 2 h to solidify and dry. Human NP cells were seeded at a density of 0.5 × 10^6^ cells per Transwell in a 24-well plate and maintained in Dulbecco’s modified Eagle’s medium (Invitrogen, CA) and 10% fetal bovine serum (Invitrogen, CA) supplemented with 2.5% Matrigel and 1% antibiotics (Invitrogen, CA) at 37 °C in a humidified incubator containing 5% CO_2_. The medium was changed twice per week.

### IVDD rat model

The IVDD rat model was established in Sprague–Dawley rats by AF needle puncture as described in our previous study^32^. In brief, general anesthesia was administered using pentobarbital 1% sodium (100 mg/kg). After performing a longitudinal incision in the supine posture, we located the renal iliolumbar vein, which was adjacent to the L4/5 intervertebral disc, and then the L3/4 intervertebral disc was visualized in the upper segment. A 21-gauge needle was inserted into the L3–L4 disc parallel to the endplates by 3.0 mm with a tiny clamp as a stopper and maintained for 30 s. No treatment was performed on rats in the control group. Subsequently, we used 3–0 silk sutures to close the muscles and 4–0 nylon sutures to close the skin margins. Finally, lumbar MRI examinations were performed on the rats 4 weeks after the operation. All procedures and protocols were approved by the Medical Ethics Committee of The First Affiliated Hospital of Sun Yat-sen University.

### RNA isolation, cDNA synthesis, and RT-qPCR

Total RNA was isolated with TRIzol reagent (Ambion, Life Technologies, Carlsbad, CA, USA) from NP tissues or cultured cells according to the manufacturer’s instructions. RNA quantity was analyzed using a Nanodrop (Thermo Scientific, USA). The mRNA was converted to complementary DNA (cDNA) using Prime Script RT Master Mix (TaKaRa). All reactions were run on a real-time PCR system (Applied Biosystems) and analyzed using the comparative Ct (ΔΔCt) method (2^−ΔΔCt^ with logarithm transformation). The following primers were used: human Grem1 (F: 5′-GAGTTGCCTTGAGAGGGTCC-3′, R: 5′-TACTCGGGGATCGGCAAATG-3′); human GAPDH (F: 5′-GACAGTCAGCCGCATCTTCTT-3, R: 5′-AATCCGTTGACTCCGACCTTC-3′); rat Grem1 (F: 5′-CGGCACTTTCCTTCGTGTTC-3′, R: 5′-GCCGTGCGATTCATTCTGTC-3′); and rat β-actin (F: 5′-ATCATTGCTCCTCCTGAGCG-3′); (R: 5′-AGCTCAGTAACAGTCCGCC-3′). GAPDH or β-actin was used for normalization.

### Immunohistochemical staining

The discs from humans and rats were fixed in 4% paraformaldehyde for 1 week, decalcified in 20% EDTA for 2–3 weeks, paraffin-embedded, and then carefully sectioned to a 7-μm thickness. After deparaffinization, antigen retrieval and blocking with 5% goat serum, the slides were incubated with Grem1 primary antibody (1:200, Abcam) and a secondary antibody. Subsequently, the sections were developed with DAB solution (GeneTech, China) and then counterstained with hematoxylin. Histological images were acquired using an Olympus BX63 microscope (Olympus, Japan) in randomly selected fields of each section at 400× magnification. The percentages of Grem1+ cells were quantified using ImageJ software (National Institutes of Health, Bethesda, MD, USA). We considered <50% positive staining as low expression of Grem1 and ≥50% positive staining as high expression of Grem1.

### Immunofluorescence analyses

Human NP cells (5 × 10^4^ cells per well) were grown on confocal plates and incubated at 37 °C. After 24 h, the cells were treated for 24 h and then fixed. Then, the cells were fixed with 4% formalin and permeabilized with 0.1% Triton X-100. After these samples were washed with PBS, the cells were blocked for 1 h with 10% goat serum (Thermo). The cells were then incubated with Grem1 antibodies (1:200, Santa Cruz) overnight at 4 °C. The cells were incubated with a fluorescent secondary antibody for 2 h at room temperature. The nuclei were stained with DAPI. Digital images of the cells were obtained using a Leica TCS SP8 microscope (Wetzlar, Germany).

### In vitro siRNA transfection

Small interfering RNAs (human Grem1 siRNA, targeting the sequence ATGAGCCGCACAGCCTACA; rat Grem1 siRNA, targeting the sequence GCAAATACCTGAAGCGAGA) were constructed by RiboBio (Guangzhou, China) and used to inhibit the expression of Grem1. Human NP cells were cultured in six-well plates to 60–70% confluence and were transfected with 50 nM negative control (NC) or Grem1 siRNA using Lipofectamine 3000 (Invitrogen) according to the manufacturer’s instructions. After 48 h, cellular lysates were obtained to analyze the expression of the genes of interest.

### Flow cytometry and TUNEL assays

NP cells were harvested with 0.25% trypsin (Gibco Life Technologies) and then stained using an Annexin V-FITC Apoptosis Detection Kit (MultiSciences) according to the manufacturer’s instructions. Briefly, NP cells were washed twice with cold PBS and resuspended in 100 µL of binding buffer at a concentration of 1 × 10^6^ cells/ml. After being stained with 5 µL of Annexin V-fluorescein isothiocyanate (FITC) and 10 µL propidium (PI) and being incubated for 5 min at room temperature, NP cells were analyzed by flow cytometry (Beckman Coulter, Fullerton, CA). We divided the total number of cells by the number of cells undergoing apoptosis to obtain the apoptotic index. NP cells grew for 24 h and were treated, and apoptosis was measured using the TUNEL assay according to the manufacturer’s protocol (Meilunbio, China). In brief, the cells were deparaffinized, rehydrated, washed with PBS, and then incubated with the TUNEL reaction mixture for 60 min at 37 °C. Finally, images were analyzed by a microscope (Wetzlar, Germany).

### Western blotting

Total protein was extracted from human NP specimens or cultured primary NP cells using RIPA buffer supplemented with protease and phosphatase inhibitors and phenylmethanesulfonyl fluoride (PMSF). The protein concentrations were measured by a BCA Protein Assay Reagent Kit (Pierce Biotechnology, Rockford, IL, USA). Total cellular proteins (20 µg per well) were loaded and resolved on 10% sodium dodecyl sulfate-polyacrylamide gels and then transferred to 0.22 µm PVDF membranes (Millipore) by electroblotting. The membranes were blocked with 5% dry skimmed milk in TBS-T for 2 h and then incubated with the following primary antibodies at 4 °C overnight: anti-Grem1 (1:1000, Abcam), anti-GAPDH (1:3000, Proteintech, China), anti-β-actin (1:3000, Proteintech, China), anti-Bax (1:1000, ZenBioScience, China), anti-Bcl-2 (1:1000, ZenBioScience, China), anti-cleaved Caspase-3 (1:1000, Cell Signaling Technology), anti-collagen II (1:1000; Abcam), anti-aggrecan (1:100; Abcam), anti-MMP3 (1:1000; Abcam), anti-p-Smad2 (1:1000, Cell Signaling Technology) and anti-p-Smad3 (1:1000, Cell Signaling Technology). After being washed with TBS-T, the membranes were incubated with HRP-linked anti-rabbit immunoglobulin G (IgG; 1:1000, Cell Signaling Technology) or HRP-linked anti-mouse IgG (1:1000, Cell Signaling Technology) for 2 h. Subsequently, the protein bands were detected with an ECL solution (ATTO Corporation, Japan). The results were quantified using a multigauge densitometry system (Fujifilm, Tokyo, Japan).

### Statistical analysis

Statistical analyses were performed using SPSS 23.0 software (IBM, USA). One-way analysis of variance and Student’s *t* test were used to analyze the differences between groups. Typically, the results are presented as the mean ± SD, and *p* < 0.05 was considered statistically significant.

## Results

### Discovery of IVDD-associated genes by microarray

**Fig. 1:**
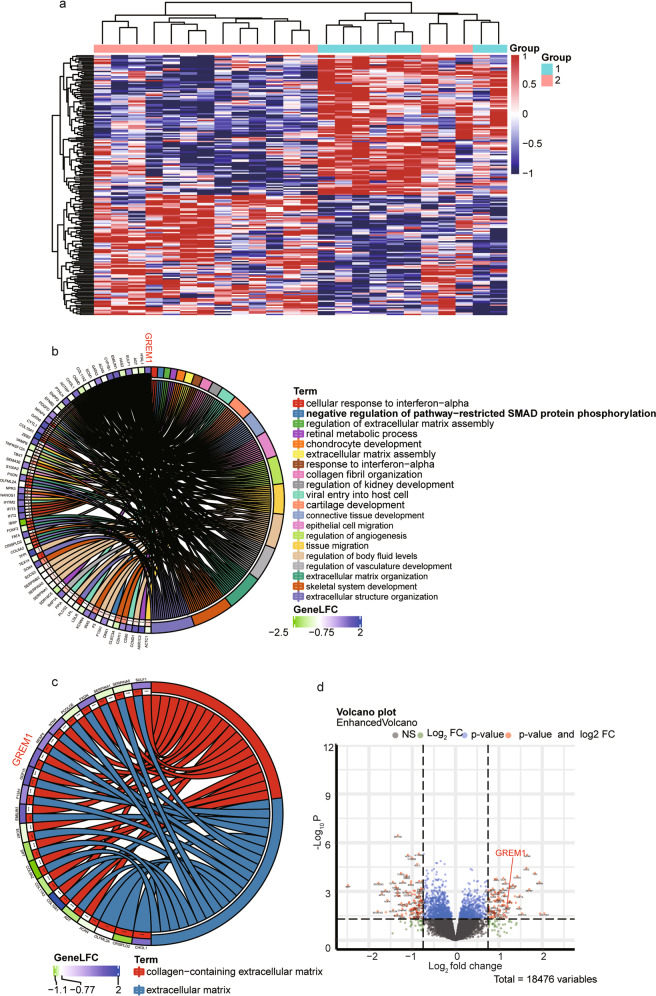
Identification of abnormally expressed genes associated with IVDD in the GSE70362 dataset. **a** Heatmap depicting all abnormally expressed genes in degenerative NP and nondegenerative NP tissues. **b**, **c** Circos plot showing differentially expressed genes. The left column represents differentially expressed genes, and the right column is different biological processes. **d** Volcano plot showing dysregulated genes.

To determine the biological roles of differentially expressed mRNAs (DEMs) in IVDD, we first examined the microarray profiles (GSE70362) of 16 degenerative NP tissue samples versus 8 nondegenerative NP tissue samplesderived from 18 donors^33^. We identified 197 significantly differentially expressed genes, including 43 upregulated DEMs and 46 downregulated DEMs, in degenerative NP compared with nondegenerative NP (Fig. 1a). By using the criterion log2-fold change values above the cutoff of ±0.75, insulin-like growth factor binding protein 3 (IGFBP3) was shown to be the most significantly upregulated gene, followed by Grem1. Moreover, we found that Grem1 was involved in more than 20 biological processes (Fig. 1b, c) and that the expression of Grem1 was positively correlated with IVDD (Fig. 1d). Because the function of IGFBP3 in IVDD has been fully studied, we evaluated whether Grem1 had disease-specific effects on IVDD.

### Upregulated expression of Grem1 in human degenerative intervertebral discs

**Fig. 2:**
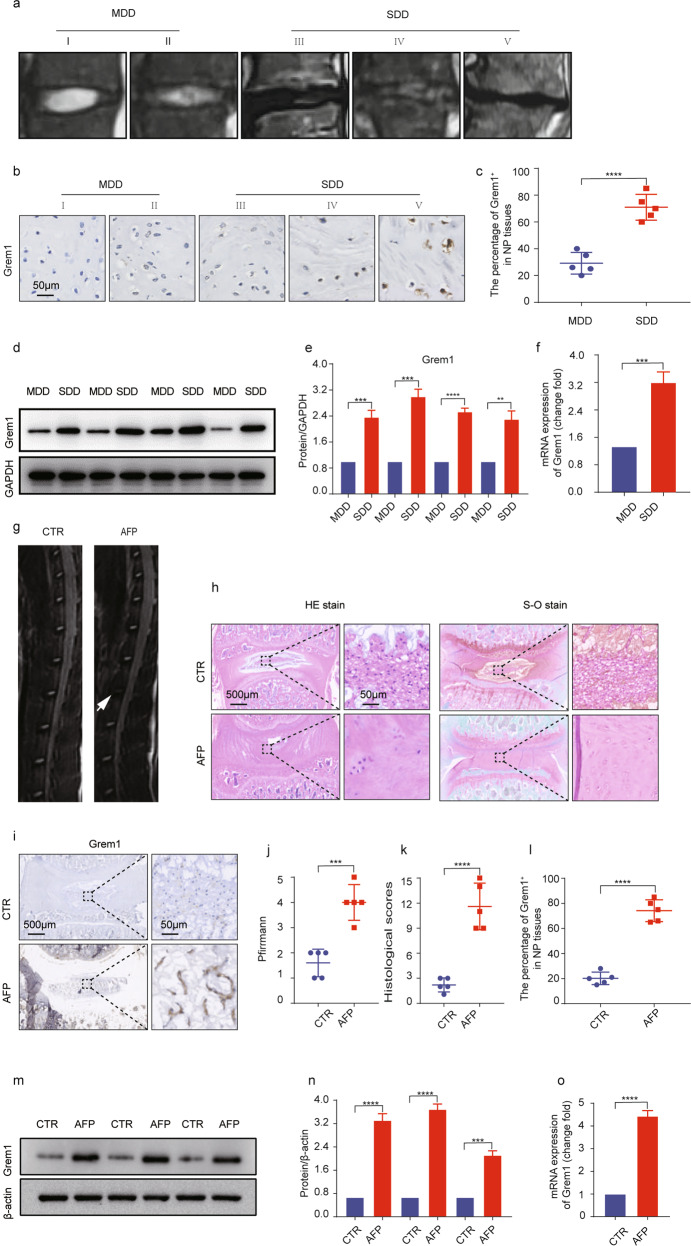
Upregulated expression of Grem1 in human degenerative and rat model intervertebral discs. **a** Representative MRI images showing the different grades of degeneration in human tissues: mild (Grades I and II) and severe (Grades III–V). **b**, **c** Grem1 was expressed in human NP tissues in the control (MDD) and SDD groups, as determined by immunohistochemistry, and quantitative analysis was performed. **d**, **e** Western blot analysis of Grem1 in disc tissues was performed. ImageJ or Image Lab software was used to determine the band intensities, which were normalized to GAPDH. **f** RT-qPCR analysis of Grem1 in MDD and SDD disc tissues. **g**, **j** Representative MRI images are shown for the control and IVDD rat models, and quantitative analysis based on the Pfirrmann grade was performed. **h**, **k** Representative images of HE staining, safranin-O staining, and histological scores of rat disc tissues are shown. **i**, **l** Immunohistochemical staining of Grem1 and qualification analysis. **m**, **n** Grem1 was expressed in rat model disc tissues, and quantitative analysis was performed. **o** RT-qPCR analysis of Grem1 in rat model disc tissues. The data are expressed as the mean ± SD (*n* = 3); *p < 0.05; **p < 0.01; ***p < 0.001; ****p < 0.0001. MDD mildly degenerated intervertebral discs, SDD severely degenerated intervertebral discs.

To investigate the role of Grem1 in IVDD, we measured the expression of Grem1 in mildly (Grades I and II) and severely (Grades III, IV, and V) degenerated human intervertebral discs. Representative MRI images showed different grades of degeneration in human tissues (Fig. 2a). As expected, immunohistochemistry (IHC) staining revealed that the expression of Grem1 was significantly increased in severely degenerated discs compared with mildly degenerated discs (Fig. 2b, c). Western blotting (WB) and quantitative analysis confirmed these findings, and lower levels of Grem1 protein were found in mildly degenerated discs (Fig. 2d, e). The real-time quantitative PCR (RT-qPCR) analysis of the mRNA expression of Grem1 was consistent with the western blot and IHC results (Fig. 2f). These results showed a positive correlation between the expression of Grem1 and the severity of IVDD.

### Increased expression of Grem1 in the IVDD rat model

To determine whether the expression of Grem1 varied in IVDD rat models of different severities, we examined certain related indicators in rat disc tissues. The MRI results confirmed that the IVDD rat models were successfully established, and there were reduced signal intensities in the AF puncture group (Fig. 2g, j). As expected, hematoxylin and eosin (H&E) staining and safranin-O (S-O) staining (Fig. 2h, k) revealed that mildly degenerated discs were composed of mucopolysaccharide-containing NP, and the AF surrounding the NP was clearly identified by abundant fibrous cartilaginous cells and an increased amount of gel-like NP tissue in the control group. However, in severely degenerated discs, the boundary between the NP and the AF gradually became indistinct, and the number of fibrous cartilaginous cells within the AF was significantly reduced. IHC and WB (Fig. 2i, l–n) showed that the expression of Grem1 was significantly higher in the AF puncture group than in the control group, and the RT-qPCR results showed the same trend in the mRNA expression of Grem1 (Fig. 2o). Taken together, these results indicated that the expression of Grem1 was elevated in degenerated discs from humans and animal models, which provided a foundation for studying the molecular mechanism of Grem1.

### TNF-α induced Grem1 expression in NP cells

**Fig. 3:**
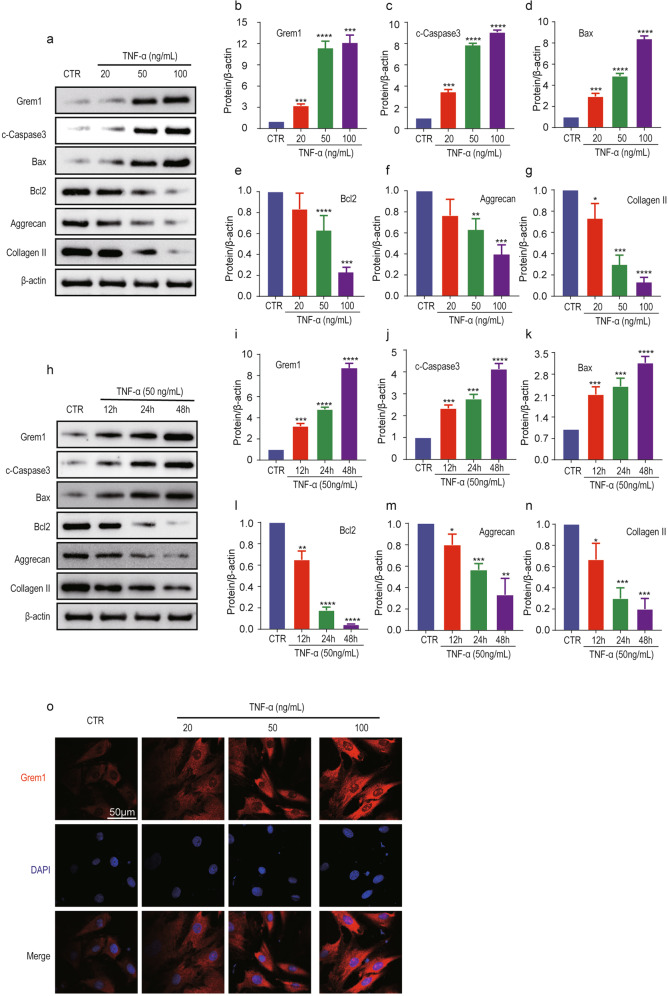
TNF-α induced Grem1 expression in NP cells. **a**, **h** Representative images of western blots showing changes in Grem1, apoptosis-related proteins (cleaved Caspase3, Bax, and Bcl2), Collagen II and Aggrecan in a dose-dependent (0, 20, 50, 100 ng/ml, 24 h) and time-dependent (50 ng/ml, 0, 12, 24, 48 h) manner in NP cells. **b**–**g**, **i**–**n** ImageJ or Image Lab software was used to determine the band intensities, which were normalized to β-actin. **o** Representative immunofluorescence images are shown to visualize the expression of Grem1 in NP cells (magnification: ×400). The data are expressed as the mean ± SD (*n* = 3); *p < 0.05; **p < 0.01; ***p < 0.001; ****p < 0.0001.

Tumor necrosis factor (TNF)-α is a cytokine that is involved in the pathogenesis of IVDD and is often used to establish a cellular degeneration model of IVD in vitro. Our previous studies indicated that inflammation could induce NP cell apoptosis; thus, we examined the expression of Grem1 and markers related to apoptosis in a TNF-α-induced NP cell model. The WB results and quantitative analysis indicated that Grem1 protein expression in NP cells was induced by TNF-α in a dose-dependent (0, 20, 50, 100 ng/ml, 24 h) and time-dependent manner (50 ng/ml, 0, 12, 24, 48 h) (Fig. 3a, b, h, i). Furthermore, the expression of the proapoptotic proteins cleaved-caspase3 and Bax was increased, and the antiapoptotic protein Bcl-2 was decreased at the same time (Fig. 3a, c–e, h, j–l). Furthermore, aggrecan and collagen II expression was dose-dependently (Fig. 3a, f, g) and time-dependently (Fig. 3h, m, n) decreased. Immunofluorescence (IF) staining further verified the results of WB and RT-qPCR (Fig. 3o). As expected, we found a positive correlation between the expression levels of apoptotic markers and the expression of Grem1.

### Grem1 siRNA prevented TNF-α-induced apoptosis-related gene expression in NP cells

**Fig. 4:**
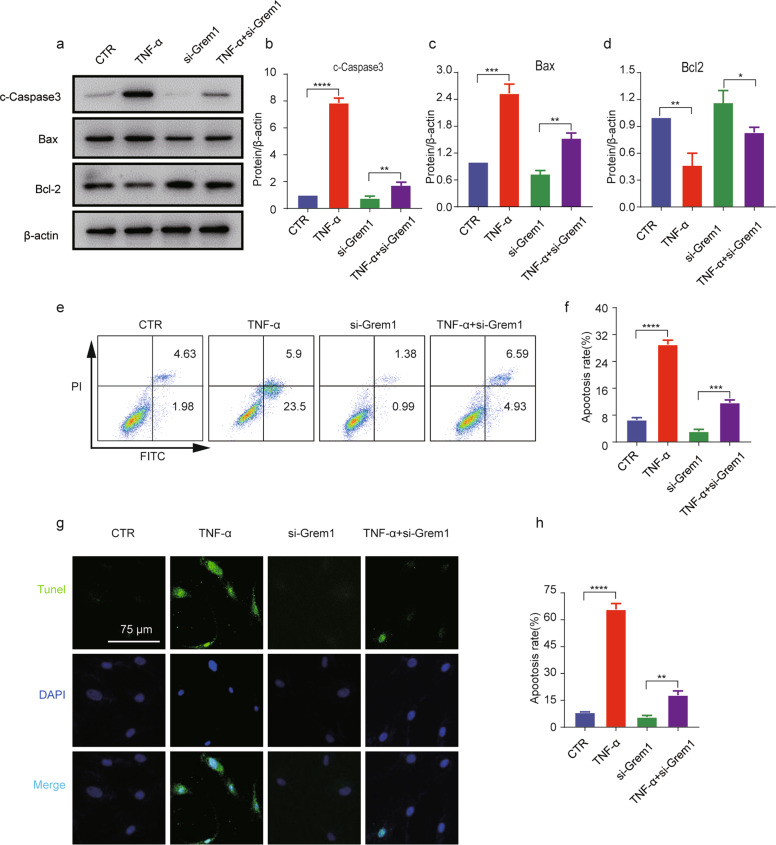
Grem1 siRNA prevented TNF-α-induced apoptosis-related gene expression in NP cells. **a** The protein expression of cleaved Caspase-3, Bax, and Bcl2 in the different treatment groups, as determined by western blotting. **b**–**d** ImageJ or Image Lab software was used to determine the band intensities, which were normalized to β-actin. **e**, **g** Representative images of flow cytometry and TUNEL staining (magnification: ×400) are shown in groups after the different treatments. **f**, **h** Quantitative analysis was performed. The data are expressed as the mean ± SD (*n* = 3); *p < 0.05; **p < 0.01; ***p < 0.001; ****p < 0.0001.

To investigate the effects of Grem1 on IVDD, we knocked down Grem1 in NP cells using siRNA. As expected, the protein levels were significantly decreased in NP cells that were transfected with Grem1 siRNA. Notably, pretreatment with Grem1 siRNA significantly suppressed TNF-α-induced cleaved Caspase-3 and Bax expression but significantly upregulated the protein levels of Bcl-2 (Fig. 4a–d). Since we confirmed that Grem1 was partly involved in the regulation of apoptosis-related genes, flow cytometry and TUNEL staining were used to determine whether Grem1 could protect NP cells from TNF-α-induced apoptosis (Fig. 4e–h). Few apoptotic NP cells were observed in the control group, and the number was significantly increased in the TNF-α-treated group. Moreover, Grem1 siRNA partly prevented TNF-α-induced apoptosis in NP cells.

### Identification of Smad-2/3 as a target gene of Grem1

To further investigate the molecular mechanisms of Grem1 in the process of IVDD, we examined the underlying signaling pathways in Grem1-mediated degeneration in IVDs in GSE70362. Gene Ontology (GO) and Kyoto Encyclopedia of Gene and Genomes (KEGG) pathway analyses of DEGs (FDR <0.05) with the R package “Cluster profile” showed that the Smad2/3 pathway and Grem1 contributed to this process (Fig. 1b). In addition, we found that Grem1 is associated with the negative regulation of Smad2/3 phosphorylation. Based on these findings, the Smad-2/3 nuclear pathway was identified as a target of Grem1.

### Grem1 regulated IVDD by suppressing the TGF-β/Smad signaling pathway

**Fig. 5:**
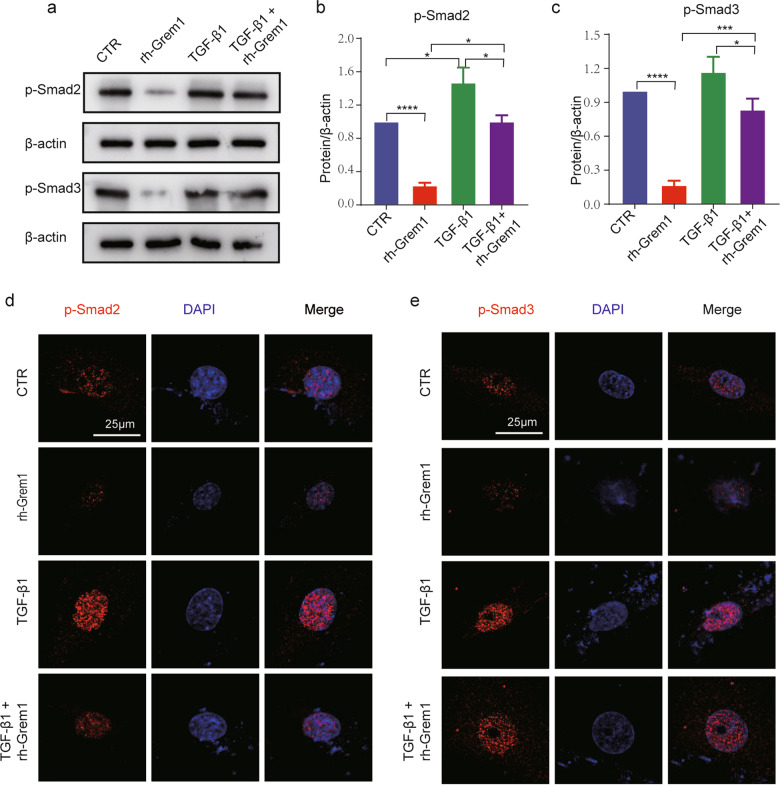
Identification of Smad-2/3 as a target gene for Grem1. **a** p-Smad2 and p-Smad3 levels after rh-Grem1 (50 ng/mL, 30 min) or TGF-β1 (20 ng/mL, 30 min) treatment, as determined by western blotting. **b**, **c** ImageJ or Image Lab software was used to determine the band intensities, which were normalized to β-actin. **d**, **e** Representative immunofluorescence images are shown to visualize the expression of p-Smad2 and p-Smad3 in NP cells (magnification: ×400). The data are expressed as the mean ± SD (*n* = 3); *p < 0.05; **p < 0.01; ***p < 0.001; ****p < 0.0001. p-Smad2 phosphorylated Smad-2. p-Smad3 phosphorylated Smad-3.

**Fig. 6:**
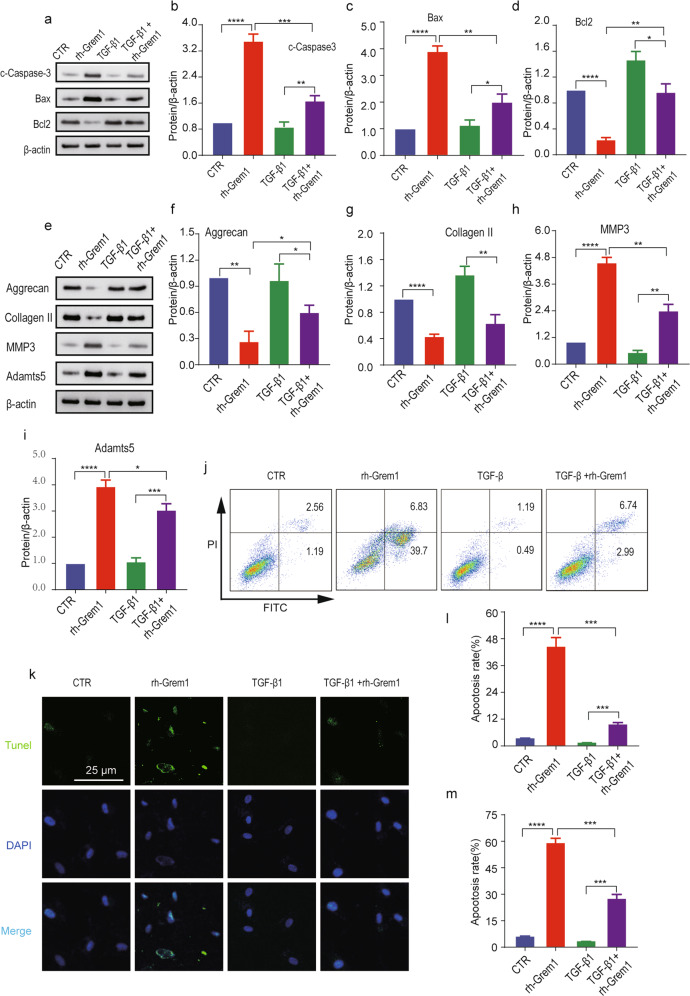
Grem1 regulates IVDD by suppressing the TGF-β/Smad signaling pathway. **a**, **e** The protein expression of cleaved Caspase-3, Bax, and Bcl2 and aggrecan, collagen II, MMP3, and Adamts5 in groups treated with rh-Grem1 (50 ng/mL, 24 h) or TGF-β1. **b**–**d**, **f**–**i** ImageJ or Image Lab software was used to determine the band intensities, which were normalized to β-actin. **j**, **k** Representative images of flow cytometry and TUNEL staining (magnification: ×400) are shown in groups after the different treatments. **l**, **m** Quantitative analysis was performed. The data are expressed as the mean ± SD (*n* = 3); *p < 0.05; **p < 0.01; ***p < 0.001; ****p < 0.0001.

According to our results, the phosphorylation of Smad-2/3 was potently inhibited in NP cells treated with recombinant human Gremlin-1 (rh-Grem1, 50 ng/mL, 30 min, Minneapolis), and the expression levels of aggrecan and collagen II were significantly suppressed in the rh-Grem1-treated group compared with the control group. To further elucidate the underlying molecular mechanisms of Grem1 in IVDD, the level of p-Smad-2/3 was measured in the rh-Grem1- or TGF-β1 (20 ng/mL, 30 min, Littleton)-pretreated groups. WB and IF analysis (Fig. 5a–e) showed that the expression levels of p-Smad-2/3 were upregulated in NP cells treated with TGF-β1 and downregulated in cells treated with rh-Grem1. Moreover, our findings demonstrated that rh-Grem1 could reverse TGF-β-mediated promotion of Smad-2/3 phosphorylation. To further explore the role of the TGF-β/Smad signaling pathway in NPC apoptosis and ECM production, we also examined markers of apoptosis and the ECM. As expected, rh-Grem1 (50 ng/mL, 24 h) decreased the expression of Bcl2 (Fig. 6a, d), aggrecan, and collagen II (Fig. 6e–g) and increased cleaved Caspase3, Bax (Fig. 6a–c), MMP3, and Adamts5 (Fig. 6e, h, i), and this effect could be partially rescued by TGF-β1. The same results were observed by flow cytometry and TUNEL staining (Fig. 6j–m). These results indicated that Grem1 regulated IVDD partially through the TGF-β/Smad2/3 pathway.

### Grem1 siRNA alleviated surgery-induced IVDD by modulating the TGF-β/Smad signaling pathway in an IVDD rat model

**Fig. 7:**
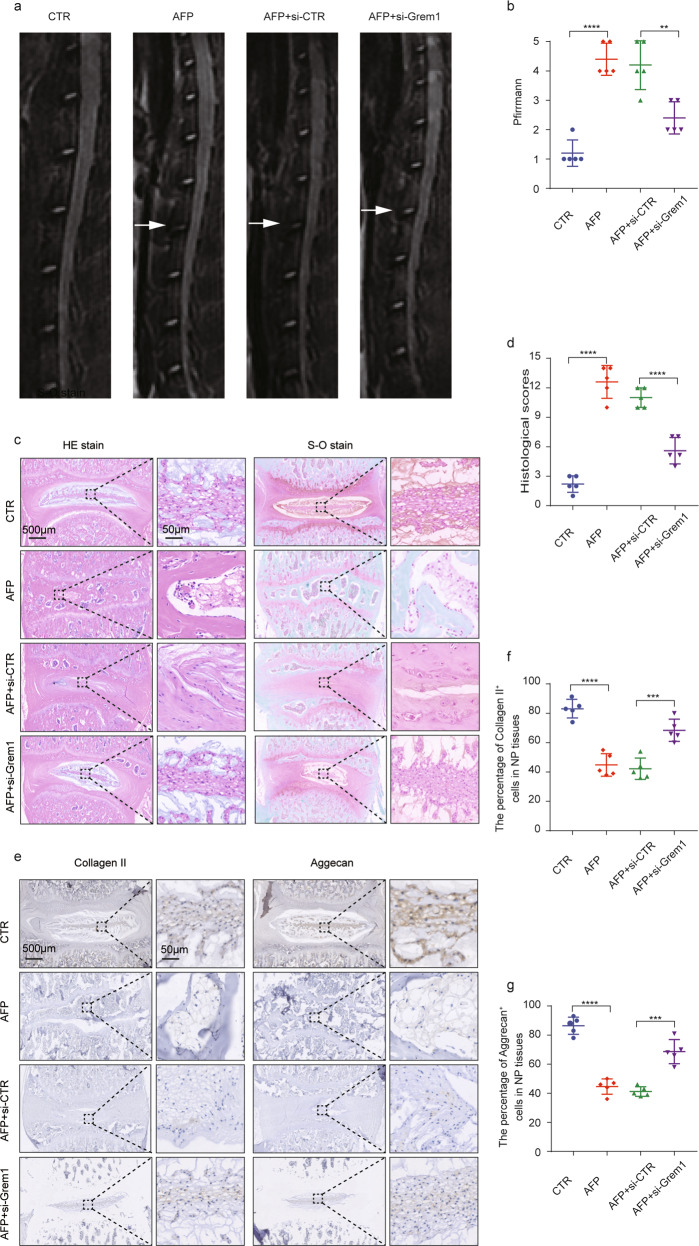
Grem1 siRNA alleviated surgery-induced IVDD in a rat model. **a**, **b** The four MRI images shown are representative of the different groups (CTR, AFP, AFP + si-CTR, AFP + si-Grem1) 8 weeks after surgery, and quantitative analysis based on the Pfirrmann grade was performed. **c**, **d** Representative images of HE staining, safranin-O staining, and histological scores in the different groups are shown. **e**–**g** Immunohistochemical staining of aggrecan and collagen II and qualification analysis are presented (magnification: ×400). The data are expressed as the mean ± SD (*n* = 5); *p < 0.05; **p < 0.01; ***p < 0.001; ****p < 0.0001. CTR control, AFP annulus fibrosus puncture, si-CTR control siRNA, si-Grem1 Grem1 siRNA.

**Fig. 8:**
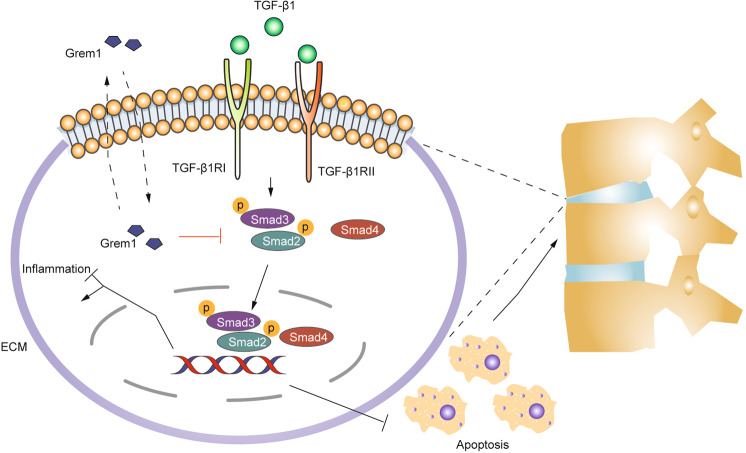
The mechanism by which Grem1 regulates the TGF-β1/Smad2/3 signaling pathway in IVDD. Grem1 inhibited Grem1-induced apoptosis and ECM component alteration by mediating the TGF-β/Smad signaling pathway. ECM extracellular matrix.

To verify the therapeutic role of Grem1 siRNA in delaying the degeneration of intervertebral discs in vivo and to clarify the underlying molecular mechanisms, we administered specific Grem1 siRNA to an AF puncture-induced IVDD rat model. The rats were divided randomly into 4 groups (5 rats per group): control (CTR), AF puncture (AFP), AFP and control siRNA (siControl (2′ OMe + 5′ chol-modified) and AFP and Grem1 siRNA (Grem1 siRNA (2′ O-methyl(OMe) + 5′ cholesterol (chol) + 5′ Cy5-modified)) (RiboBio). Grem1 siRNA and the control were injected into L3/4 IVDs (5 nmol; 10 µL) at 1, 7, and 14 days after surgery. MRI was performed 8 weeks after surgery (Fig. 7a), and the results showed that the Pfirrmann grade scores of the Grem1 siRNA group were significantly lower than those of the AF and control siRNA groups (Fig. 7b). This result suggested that Grem1 siRNA could slow the progression of IVDD in vivo. Consistent with the imaging results, histological observations (Fig. 7c, d), including H&E and S-O staining, revealed a relatively indistinct boundary between the NP and the AF in the IVDD rat model and the control siRNA group, and this effect was alleviated in IVDD rat models treated with Grem1 siRNA. Likewise, the IHC results suggested that the expression levels of Collagen II and Aggrecan were reversed in the Grem1 siRNA group compared with the IVDD rat model or control siRNA group (Fig. 7e–g). Overall, these results indicated the therapeutic potential of suppressing Grem1 to alleviate IVDD (Fig. 8).

## Discussion

LBP is an urgent global public health concern that has a negative impact on quality of life^34^. Treatment modalities for IVDD include conservative and surgical strategies, but it is difficult to successfully address the underlying biological problem^35^. Therefore, novel and stage-adjusted therapies to combat IVDD are urgently needed. Accumulating evidence has shown that Grem1 can induce inflammation and is implicated in many pathogenic mechanisms and diseases, such as heart, lung, and liver fibrosis, as well as osteogenesis, angiogenesis, and cancer^12,14,36^. It is worth noting that inflammation coupled with imbalanced ECM synthesis is widely recognized as a key characteristic related to the development and progression of IVDD^7,8^. In this study, we first identified Grem1 as a key gene involved in IVDD at the cellular, tissue, and organism levels. Grem1 participated in the TNF-α-induced proapoptotic response and imbalanced anabolism and catabolism of the ECM. Moreover, we also found that rh-Grem1 could promote apoptosis and ECM degradation. Then, the TGF-β1/Smad signaling pathway was identified as a target of Grem1 by GO analysis. Next, we studied the effects of Grem1 on mediating the TGF-β1/Smad signaling pathway in NPCs. The results showed that Grem1 could inhibit the phosphorylation of Smad2/3, while TGF-β1 promoted the phosphorylation of Smad2/3. Moreover, TGF-β1 partially reversed rh-Grem1-mediated inhibition of p-Smad2/3 expression in NPCs and consequently decreased NPC apoptosis and ECM catabolism induced by rh-Grem1 expression. Similarly, Grem1 disrupted TGF-β1-mediated promotion of Smad2/3 phosphorylation. Therefore, TGF-β1 reversed the effects of Grem1 on inducing apoptosis, inhibiting ECM anabolism, and promoting ECM catabolism by activating the TGF-β1/Smad signaling pathway and consequently repressing the progression of IVDD. Furthermore, these conclusions were verified by in vivo experiments.

Grem1, which is a member of the DAN (differential screening-selected gene aberrative in neuroblastoma) family of secreted BMP antagonists, selectively binds to BMP-2, -4, and -7 to exert biological functions^37^. Grem1 is believed to have a variety of functions, such as regulating the development of the skeleton, kidney, retina, and lung, and is involved in the pathogenic mechanisms of lung and liver fibrosis, osteogenesis, angiogenesis, and cancer^12,14,38^. In addition to its inhibitory effects on BMPs, Grem1 also affects cell functions via BMP-independent mechanisms^12,14^. In this study, Grem1 was identified as one of the top upregulated DEMs in NP tissues from IVDD patients using a microarray. Numerous studies have confirmed that Grem1 plays a key role in OA^17,19^. However, the distinct role of Grem1 in regulating the progression of IVDD remains unclear. Previous studies have shown that Grem1 is expressed in NPCs^21,22^, which was confirmed in this study, and we found that Grem1 was upregulated in degenerated cells, samples, and animal models of IVDD. Furthermore, using RT-qPCR, WB, and IHC to analyze an appropriate number of patient NP samples, we demonstrated that the expression level of Grem1 was positively correlated with the severity of disc degeneration. Grem1 was significantly overexpressed in TNF-α-induced degenerative NPC models. Apoptosis in degenerative NP cell models that were transiently transfected with siRNA targeting Grem1 was significantly lower than that in the control group. Of note, Grem1 siRNA effectively alleviated IVDD in a surgery-induced IVDD rat model. Collectively, these findings indicate that Grem1 is a potential therapeutic target for IVDD.

TGF-β belongs to the TGF-β superfamily, which includes TGF-βs, activins, BMPs, and related proteins^39^. TGF-β1 is the most abundant and studied isoform, and the other isoforms are TGF-β2 and TGF-β3. These proteins were identified mainly depending on their roles in regulating tissue homeostasis, embryonic development, and regeneration^40^. Accordingly, dysfunction of TGF-β family members has been implicated in cancer, fibrosis, immune diseases, and many other pathologies, including proliferation, differentiation, ECM balance, apoptosis, and senescence^39–42^. The TGF-β signaling pathway plays a key and unique role during the development and progression of OA^26^. Chen et al. examined TGF-β signaling in intervertebral disc health and disease and concluded that TGF-β signaling pathway signaling inhibited ECM degradation, increased ECM synthesis, promoted cell proliferation, inhibited cell death, and alleviated the inflammatory response^29^. As a canonical signaling pathway, TGF-β binds to its receptor complex and promotes the phosphorylation of Smad2 and Smad3, followed by activating R-Smads and forming trimeric complexes with Smad4^43^. The activation of TGF-β signaling in cartilage maintains cell phenotype and tissue homeostasis through the Smad2/3 pathway^44^, resulting in ligand-induced transcription that upregulates the expression of Aggrecan, Col2, TIMP-3, and Sox9^29^. Our previous study showed that TGF-β1 protected against IVDD by inhibiting syndecan-4 expression^45^. The structure of Grem1 contains a cysteine knot motif and a structure that is shared by members of the TGF-β superfamily^15^. We have clarified the roles of Grem1 in IVDD, and we suggest that Grem1 participates in the phosphorylation of Smad2 and Smad3. Moreover, one study predicted that Grem1 could bind to the TGF-β1 protein, which fits our hypotheses and needs further verification^23^. In the present study, we indicated that Grem1 regulates IVDD partially by inhibiting the TGF-β1/Smad2/3 signaling pathway by promoting apoptosis and altering ECM components.

Previous studies attributed the regulation of inflammation-induced apoptosis and ECM component alterations to multiple factors related to the TGF-β superfamily. We clarified that the inflammatory response played a key role in IVDD^10,46–49^, where TGF-β1 was involved in regulation^45^. In this study, we explored the relationship between Grem1 and inflammatory reactions in NPCs and found that the expression of Grem1 was upregulated in NPCs treated with TNF-α. Therefore, we hypothesized that Grem1 regulated IVDD by inhibiting the inflammatory response, and further investigations are needed to confirm this hypothesis.

Although the therapeutic effect of Grem1 siRNA on a surgery-induced IVDD rat model was beneficial, the optimal dose remains to be determined, and the side effects are still unknown. There are still several problems to be solved, and many hurdles need to be overcome before this treatment is administered to humans. Moreover, the precise mechanism by which the Grem1 level increases during the degenerative process remains unclear. Further studies are required to completely elucidate the role of Grem1 in IVDD.

In conclusion, our findings indicate that the expression of Grem1 is positively correlated with the severity of IVDD and demonstrate that local delivery of a specific Grem1 siRNA observably alleviates IVDD in a surgery-induced IVDD rat model. For the first time, our research links Grem1 with the TGF-β/Smad signaling pathway and demonstrates the molecular mechanisms regulating the maintenance and destruction of IVDs. In brief, our results provide a potential therapeutic target for alleviating NPC apoptosis and inflammatory-induced ECM component alterations associated with IVDD.

## Data Availability

All data from this study are included within the article.
